# Case report: A case of renal vessel rupture caused by severe vomiting in early pregnancy

**DOI:** 10.1097/MD.0000000000039025

**Published:** 2024-07-26

**Authors:** Jiang Deng, He Huang, Jinhua Ma, Hongbing Wei, Xi Qu, Zheng Hu, Hui Zeng, Jun Zhou

**Affiliations:** 1 a Hubei No. 3 People’s Hospital of Jianghan University, Wuhan, China.

**Keywords:** case, pregnancy, renal vessel rupture

## Abstract

**Objective:**

Spontaneous renal vessel rupture is a rare clinical emergency. However, pregnancy symptoms and signs are not obvious, and the limited examination methods obscure the observation. Thus, early renal rupture is challenging to detect, leading to misdiagnosis and poor prognosis. This paper aims to improve clinicians’ understanding of this disease and reduce the rate of clinical misdiagnosis.

**Patient concerns:**

The patient, aged 23 and 11 weeks pregnant, developed severe right lumbar and abdominal pain for 14 hours after severe nausea, vomiting, and paroxysmal intensification. Color ultrasound of the urinary system at another hospital revealed right kidney stones and right ureter dilation. Thus, the patient came to our hospital for treatment.

**Diagnoses:**

Spontaneous renal vessel rupture.

**Interventions:**

In this case, the diagnosis of spontaneous renal vascular rupture and hemorrhage was confirmed. Following conservative treatment such as fluid replenishment, blood transfusion, and hemostasis, the patient was given an emergency renal artery embolization due to unstable hemodynamics during treatment and poor conservative treatment effect.

**Outcomes:**

Nephrectomy was performed after 1-week follow-up for renal necrosis.

**Lessons:**

To avoid missed diagnosis and misdiagnosis, patients with abdominal pain caused by severe vomiting during pregnancy must be closely monitored. Additionally, treatment should be considered individually to ensure the safety of both mother and child. Therefore, spontaneous renal vessel rupture should be considered as the differential diagnosis.

## 1. Introduction

Spontaneous renal artery rupture refers to the rupture of renal parenchyma, renal pelvis collection system, or renal vessels in the absence of obvious trauma. Notably, this is rare in the clinic and was first reported by Wunderlich in 1856; thus, it is also called Wunderlich syndrome.^[1]^ Spontaneous renal artery rupture is primarily pathological and can be divided into renal parenchymatous lesions, such as renal tumor, tuberculosis, or polycystic kidney; renal vascular diseases, such as renal vascular embolism and nodular arteritis; obstruction of the renal pelvis collecting system.^[2–4]^ Additionally, diagnosis of spontaneous renal artery rupture is difficult because the signs and symptoms may be similar to severe pyelonephritis, uterine rupture, kidney stones, or appendix rupture in the early stages, making it easy to misdiagnose. In this report, we present a case of spontaneous renal artery rupture in early pregnancy misdiagnosed as lithiasis.

## 2. Clinical data

A 23-year-old female patient was admitted to hospital on May 10, 2023, with persistent right back pain for 14 h. She was 11 week pregnant with 1 pregnancy and 0 delivery. There was no trauma, hypertension, or vaginal bleeding during pregnancy. Fourteen hours before admission, following severe nausea and vomiting, severe pain was noted in the right waist and abdomen with sudden occurrence, including worsening paroxysms. There were no symptoms such as pale face, cold sweat, abdominal distension, frequent urination, urgent urination and pain, etc. Color ultrasound in the outside hospital showed an abnormal shadow in the right kidney region. Hydronephrosis, renal calculus, right ureteral dilatation: After symptomatic treatment such as spasmolysis and infusion, the symptoms were not significantly relieved, and the emergency department was admitted to the urology department. The patient denied any history of urinary surgery or abdominal trauma. Coagulation results before admission: prothrombin time was measured at 14.90 seconds ↑, prothrombin time polyactivity 66.70% ↓; Renal function: carbon dioxide 15.1 mmol/L ↓, glomerular filtration rate 80.37 mL/min ↓; Blood routine WBC 16.32 × 10^9^/L, hemoglobin 101 g/L. Physical examination after admission: Body temperature 36.5°C pulse 141 times/min, breathing 23 times/min, Blood pressure 127/88 mm Hg, blood oxygen saturation: 99%. Moreover, abdominal distension was noted, with no bulge in both renal areas, mild tenderness in the right upper abdomen, tenderness, and percussion pain in the right renal area were positive, no tenderness in the right upper ureter, no mass was detected in the right ureter strike area. Furthermore, there was no filling or tenderness in the suprapubic bladder area and no redness or secretion at the external urethral orifice.

**Figure 1.**
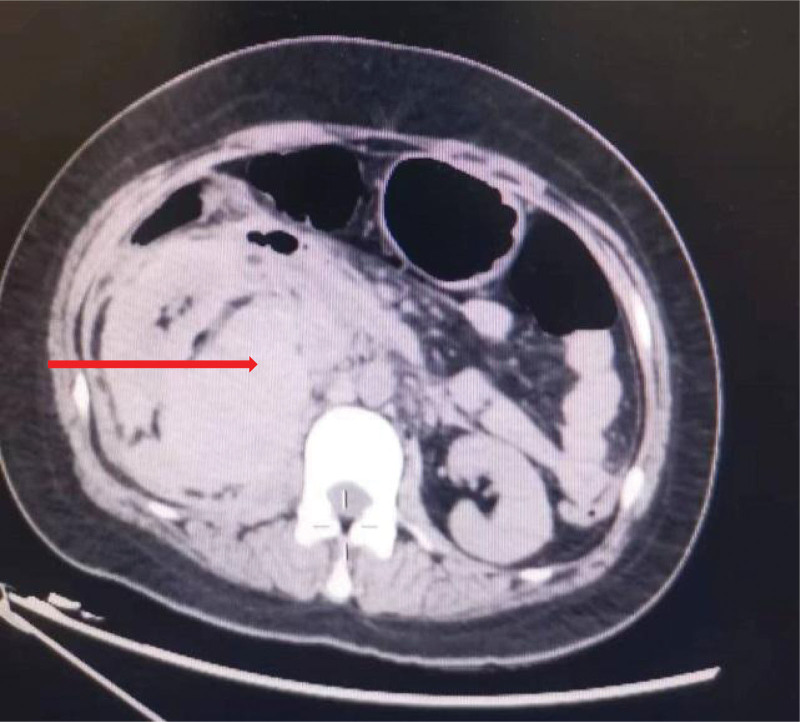
Abdominal plain scan CT shows possible rupture of the right kidney and exudation (exudation involves retroperitoneum and pelvic cavity). The arrow shows the right perirenal hematoma (size about 5.0 × 7.8 × 8.8 cm).

**Figure 2.**
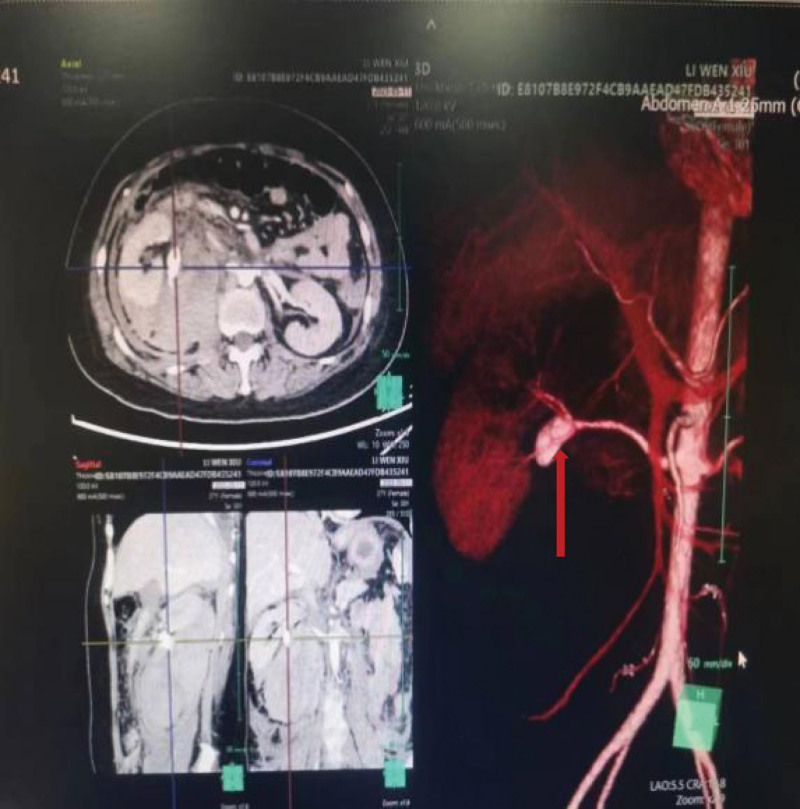
Abdominal enhanced CT + 3D imaging: rupture of the beginning of the inferior trunk branch of the right renal artery and formation of a peripheral hematoma (involving perirenal, retroperitoneal, and pelvic cavity).

Upon admission, symptomatic treatments such as finger oxygen monitoring, anti-infection, spasmolytic, analgesic, and strict absolute bed rest were given. The patient’s symptoms were not well relieved. For further diagnosis, an abdominal plain scan CT was performed 4 hours after admission (Fig. 1), indicating the possibility of rupture and exudation of the right kidney (involving retroperitoneum and pelvic cavity) and right perirenal hematoma (5.0 × 7.8 × 8.8 cm in size). Uterine changes, combined with clinical consideration of pregnancy status. Upon reexamination of blood routine: WBC 13.48 × 10^9^/L, hemoglobin 75 g/L, renal function: creatinine 129.11 µmol/L, glomerular filtration rate: 48.96 mL/min. The patient exhibited a rapid decrease in hemoglobin and was given an infusion of suspended red blood cells 2µ. Hemoglobin was 77 g/L following transfusion. Abdominal enhanced CT (Fig. 2), performed 18 hours later, showed that the beginning of the inferior trunk branch of the right renal artery was ruptured. Additionally, a peripheral hematoma formed (involving the perirenal, retroperitoneal, and pelvic cavity), which was larger than the previous film. Moreover, a large amount of intra-abdominal fluid and a small amount of bilateral pleural fluid were increased. Following conservative treatment with blood transfusion and fluid rehydration, the patient had hemodynamic instability, and emergency renal artery embolization was performed. CT examination 1 week after surgery revealed renal necrosis and a right nephrectomy was performed.

## 3. Discussion

Spontaneous renal vessel rupture is a rare surgical emergency in urology. Notably, clinical manifestations are related to the amount of blood loss and the onset of the disease. Moreover, these manifestations are mainly acute and cause persistent lumbago pain on 1 side, accompanied by nausea and vomiting. If the renal pelvis is broken, gross hematuria can be observed, severe hematuria forms blood clots blocking the ureter, and the pain is further aggravated. The signs and symptoms of spontaneous renal vessel rupture in clinical practice are similar to those of many diseases, Such as severe pyelonephritis, uterine rupture, kidney stones, or appendix rupture. Importantly, B-ultrasound has the advantages of being noninvasive, safe, and convenient during pregnancy. Early B-ultrasound has become the preferred examination method during pregnancy. However, it has limitations in identifying blood clots and tumors caused by renal vascular diseases. CT is highly diagnostic of renal vascular rupture. Enhanced CT can not only show the lesion site but also determine the scope and degree of bleeding in spontaneous renal hemorrhage caused by renal tumor and renal vascular disease, and the etiological diagnosis rate can be as high as 90%.^[5]^ In this case, the patient had persistent right lumbar pain before admission, and a color Doppler ultrasound showed an abnormal shadow in the right kidney area, right hydronephrosis, and right ureter dilation. Due to the patient being pregnant, CT and enhanced CT were not considered as the primary examination of clinicians for patients who want to preserve the fetus, which brings specific difficulties for early differential diagnosis. Previous studies have also shown that the incidence of renal vessel rupture generally has a cause, such as Hu and Mao.^[6]^ In A detailed analysis of 206 cases of spontaneous renal rupture, 63.6% were renal tumors, 15.0% were vascular diseases, 8.3% were inflammatory reactions, and 5.3% were coagulation disorders. Zhang Jianqing also reported a 20-year-old patient with renal pelvis rupture and perirenal hemorrhage caused by severe vomiting after drinking.^[7]^ In 2021, a case of spontaneous rupture of renal artery pseudoaneurysm in hemodialysis patients was reported.^[8]^ However, there are few reports on spontaneous renal vessel rupture during pregnancy, and some reports show that the risk of renal vessel rupture may be increased due to the increase in circulating blood volume and renal blood flow during pregnancy, as well as the increase of internal abdominal pressure and severe vomiting in early pregnancy.^[9]^ Regardless, the specific mechanism of spontaneous renal rupture during pregnancy is still unclear. As such, the diagnosis is complex, and the risk of renal vascular rupture is increased with severe vomiting in early pregnancy.^[9]^ Furthermore, the symptoms and signs of pregnancy are not obvious, the examination methods are limited, and early renal rupture is difficult to detect, leading to misdiagnosis and poor prognosis. Therefore, clinicians must be alerted to this extremely rare and instructive disorder.

## 4. Conclusion

The patient was in the early stage of pregnancy and had no previous history of tumor disease, congenital vascular abnormalities, coagulation abnormalities, and abdominal trauma. She was accompanied by severe and persistent nausea and vomiting before the onset of the disease. Considering the increase in blood flow during pregnancy, severe nausea and vomiting caused a further increase of lumbar and abdominal pressure to spontaneous rupture of lower blood vessels in the right kidney. Likewise, the means of examination during pregnancy were limited. Color ultrasonography failed to distinguish stones from renal vessel rupture, leading to misdiagnosis and poor prognosis. According to the diagnosis and treatment characteristics of this case, attention should be paid to the need for timely intervention in early pregnancy accompanied by persistent severe nausea and vomiting, reducing abdominal pressure, and timely intervention in pregnancy reactions. During the diagnosis and treatment of abdominal pain in pregnancy, the medical history should be carefully asked and should not be limited to common diseases and frequently occurring diseases. Moreover, diagnostic ideas should be open, especially for the particular population during pregnancy. The diagnosis and treatment process should be carefully and comprehensively considered to achieve early detection and treatment, and serious complications such as kidney necrosis should be avoided as much as possible. Thus, our experience has provided valuable insight for future cases of similar severity.

## Acknowledgments

The authors thank AiMi Academic Services (www.aimieditor.com) for English language editing and review services.

## Author contributions

**Data curation:** Jiang Deng, Jinhua Ma, Zheng Hu, Hui Zeng.

**Investigation:** Jiang Deng, Xi Qu, Zheng Hu, Hui Zeng.

**Project administration:** Jiang Deng, He Huang, Jinhua Ma, Hongbing Wei.

**Supervision:** He Huang, Jun Zhou.

**Writing – original draft:** Jiang Deng.

**Writing – review & editing:** Jiang Deng, Jun Zhou.
